# Time dependency of automated collateral scores in computed tomography angiography and computed tomography perfusion images in patients with intracranial arterial occlusion

**DOI:** 10.1007/s00234-022-03050-4

**Published:** 2022-09-27

**Authors:** Jiahang Su, Lennard Wolff, Pieter Jan van Doormaal, Diederik W.J. Dippel, Wim van Zwam, Wiro J Niessen, Aad van der Lugt, Theo van Walsum

**Affiliations:** 1 Department of Radiology & Nuclear Medicine, Erasmus MC, Rotterdam, The Netherlands; 2 Department of Neurology, Erasmus MC, Rotterdam, The Netherlands; 3 Department of Radiology, Maastricht UMC +, Maastricht, The Netherlands; 4 Faculty of Applied Science, Delft University of Technology, Delft, The Netherlands

**Keywords:** Ischemic Stroke, Collateral score, CTA, CTP

## Abstract

**Purpose:**

The assessment of collateral status may depend on the timing of image acquisition. The purpose of this study is to investigate whether there are optimal time points in CT Perfusion (CTP) for collateral status assessment, and compare collaterals scores at these time points with collateral scores from multiphase CT angiography (mCTA).

**Methods:**

Patients with an acute intracranial occlusion who underwent baseline non-contrast CT, mCTA and CT perfusion were selected. Collateral status was assessed using an automatically computed Collateral Ratio (CR) score in mCTA, and predefined time points in CTP acquisition. CRs extracted from CTP were correlated with CRs from mCTA. In addition, all CRs were related to baseline National Institutes of Health Stroke Scale (NIHSS) and Alberta Stoke Program Early CT Score (ASPECTS) with linear regression analysis to find the optimal CR.

**Results:**

In total 58 subjects (median age 74 years; interquartile range 61–83 years; 33 male) were included. When comparing the CRs from the CTP vs. mCTA acquisition, the strongest correlations were found between CR from baseline mCTA and the CR at the maximal intensity projection of time-resolved CTP (*r* = 0.81) and the CR at the peak of arterial enhancement point (*r* = 0.78). Baseline mCTA-derived CR had the highest correlation with ASPECTS (*β* = 0.36 (95%CI 0.11, 0.61)) and NIHSS (*β* = − 0.48 (95%CI − 0.72, − 0.16)).

**Conclusion:**

Collateral status assessment strongly depends on the timing of acquisition. Collateral scores obtained from mCTA imaging is close to the optimal collateral score obtained from CTP imaging.

## Introduction

Ischemic stroke caused by a proximal occlusion in the intracranial cerebral arteries normally results in poor clinical outcome [1]. Endovascular treatment (EVT), in which the intracranial thrombus is removed, improves functional outcome [2]. The collateral circulation in the presence of an intracranial occlusion is an important determinant of clinical outcome and might also influence the beneficial effect of EVT [3, 4, 5, 6, 7]. However, accurate assessment of collateral status can be challenging from two perspectives. First, the current existing semi-quantitative collateral grading systems are based on visual scoring using coarse classification criteria [8, 9] which are subject to inter-observer variation. Automatic analysis could potentially overcome this limitation [10, 11, 12]. Secondly, the collateral grading is affected by the timing of image acquisition in relation to the start of contrast media injection [13]. For an accurate prediction of treatment outcome and clinical decision-making it is relevant to know which acquisition time is optimal for assessment of collateral score.

For collateral status assessment, single-phase Computed Tomography Angiography (sCTA) is commonly used [8, 14, 15]. sCTA comes with the risk of incorrect collateral grading due to inappropriate scan timing [4] . To avoid this potential bias caused by incorrect timing, additional temporal CTA acquisitions can be added to the sCTA. The two most well-known imaging techniques are multiphase CTA (mCTA) and CT perfusion (CTP). Multiphase CTA consists of two extra acquisitions with short delays of 6–8 s between the acquisitions [16]. CTP consists of a series of acquisitions with 1.5- to 2-s interval and a total length of approximately 1 min. In previous studies, a visual collateral scoring system based on mCTA was proven to be superior to sCTA in clinical outcome prediction [13, 16].

In this study, we applied a previously developed automated collateral scoring algorithm for CTA data also to CTP data and investigated how collateral status assessment depends on the timing of image acquisition. Additionally, we extracted collateral scores at specific time points from the CTP data, and investigated how these CTP collateral scores relate to mCTA collateral scores. Finally, we assessed the association of collateral scores from CTP and mCTA with baseline ASPECTS [17] and NIHSS [18].

## Materials and methods

### Patient selection

We used data from acute ischemic stroke patients with a large vessel occlusion from Erasmus MC, who were included in the Multicenter Randomized Clinical Trials of Endovascular Treatment of Acute Ischemic Stroke (MR CLEAN LATE, MR CLEAN NOIV or MR CLEAN MED studies from January 2018 to March 2020). Detailed information on inclusion criteria, description of variables and the methods of the three trials has been reported previously [19, 20, 21]. For this study, we selected patients with the following inclusion criteria: an intracranial occlusion on one side only, baseline imaging that contains both non-contrast enhanced CT (NCCT), mCTA and CTP; slice thickness ≤ 1.5 mm for both mCTA and CTP; complete CTP acquisition (> 55 s), and absence of large motion artifact(s) in both mCTA and CTP. Motion artifacts were visually checked by the CTP drived time-invariant CTA image, which was generated by the maximal intensity projection over time after spatial alignment. The CTP image was excluded if this image had visible motion artifacts. Neurologic deficit at baseline was assessed with the National Institutes of Health Stroke Scale (NIHSS; range 0–42, with higher scores indicating more severe neurologic deficits).

### Imaging protocol

All included subjects underwent a standardized imaging protocol for acute ischemic stroke. This protocol consists of NCCT, CTP and mCTA (Siemens SOMATOM Definition Edge). The CTP image acquisition was performed with a fixed tube voltage of 70 kVp and an injection of 40 ml of Iomeron 400 with a saline bolus chaser at a flow rate of 6 ml/s. The CTP images were reconstructed with a slice thickness of 1–1.5 mm. A CTP acquisition had a total duration of 55 to 60 s and consisted of 30(± 1) volumes. After the CTP, the mCTA acquisition was performed with an automatic tube voltage of 80–100 kVp, injection of 60 ml of Iomeron 400 with a 40 ml saline bolus chaser at a flow rate of 4 ml/s. Timing of scanning was based on bolus tracking. The mCTA consists of three image volumes acquired with delays between the volumes of 8 s on average. The first acquisition of mCTA was defined as the mCTA baseline. All CTA images were reconstructed with a slice thickness of 0.75 mm and an increment of 0.4 mm.

### Automatic collateral scoring and arterial and venous enhancement curves

To obtain a consistent location for arterial and venous structures and enhancement measurements over time, all CTP image volumes were rigidly aligned to the first acquisition using registration software ANTs [22, 23]. The automatic collateral scoring algorithm was developed by [12] with collateral ratio (CR) as output (expressed in percentage %). This method was comparable to the visual collateral score obtained from 29 rater [24]. The proposed CR ranges from 0 to 150. In this method, the middle cerebral artery (MCA) region was defined with a two-step atlas based registration using ANTs [22, 23] with initial rigid registration and a subsequent diffeomorphic non-rigid registration. Vessels were identified using a U-net based vessel segmentation algorithm. The enhanced vessel volume in the occluded and contralateral MCA region was computed. In the last step, the CR was calculated as enhanced vessel volume in the MCA region of the occluded side divided by the contrast enhanced vessel volume in the healthy side.

Arterial and venous enhancement in the CTP and mCTA images was obtained by annotating a small region of interest at the top of the intracranial carotid artery (ICA) (arterial) of the non-occluded side and the confluence sinus (venous). The arterial and venous enhancement value were determined using the median image intensity of a spherical region at the locations mentioned above.

### NCCT analysis

Baseline Alberta Stoke Program Early CT Scores (ASPECTS) [17] were assessed on baseline CT images. ASPECTS was rated by radiologists with 5 to 20 years of experience. The raters were blinded to all patient information during ASPECTS evaluation, except occlusion side on baseline CTA.

### CTP time-intensity curve and feature extraction

**Fig. 1:**
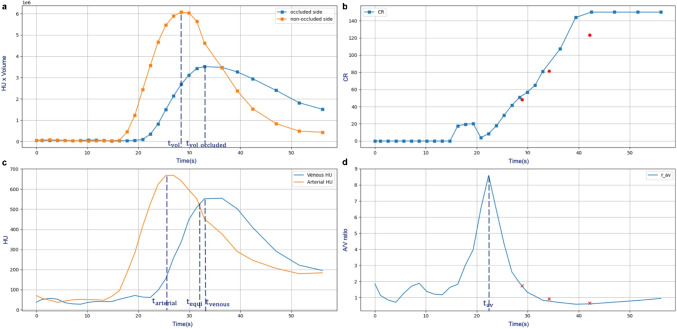
An example feature plot. (a) the contrast volume changing over time for both occluded side and contralateral side; (b) the collateral ratio changing over time, three red dots are the corresponding collateral ratios of the mCTA; (c) the arterial and venous enhancement curve; (d) the arterial to venous (AV) ratio curve, the three crosses stand for the alignment points based on AV ratios of the three mCTA acquisitions

To investigate the temporal relationship of enhanced vessel volume, CR, and arterial and venous enhancement in CTP images, four types of time-intensity curves (TIC) were generated: contrast enhanced vessel volume in the occluded and contralateral side (Fig. 1a), CR (Fig. 1b), arterial and venous enhancement (Fig. 1c) and arterial to venous (AV) enhancement ratio (Fig. 1d). In the AV enhancement ratio curve generation, in order to quantify the effect of the contrast agent, an average density value of unclotted blood (40 HU) was subtracted from the arterial and venous density values before calculating the ratio.

From these curves, the following time points were defined to extract a CR: the peak of vessel volume in the non-occluded side ($CR_{vol\_max}$); the peak of vessel volume in the occluded side ($CR_{vol\_occluded\_max}$); the peak of arterial enhancement ($CR_{arterial\_max}$); the peak of venous enhancement ($CR_{venous\_max}$); the point where the arterial enhancement equals venous enhancement (*CR*_*equi*_) and the peak of AV enhancement ratio ($CR_{av\_max}$). Also, the time between contrast arrival time and these time points was calculated. In addition, in CTP images, a time-resolved variable *CR*_*mip*_ was obtained from the timing-invariant CTA image [25]. The timing-invariant CTA was obtained using the 3D maximal intensity projection(MIP) over time from all CTP acquisitions.

### mCTA image feature extraction

For the three mCTA volumes, the CR was computed using the automatic collateral scoring method whereas the arterial and venous enhancement values were obtained with manual annotation. The AV enhancement ratio was obtained using the same approach as described for the CTP images.

### Alignment of mCTA acquisitions to the CTP and across subjects

**Fig. 2:**
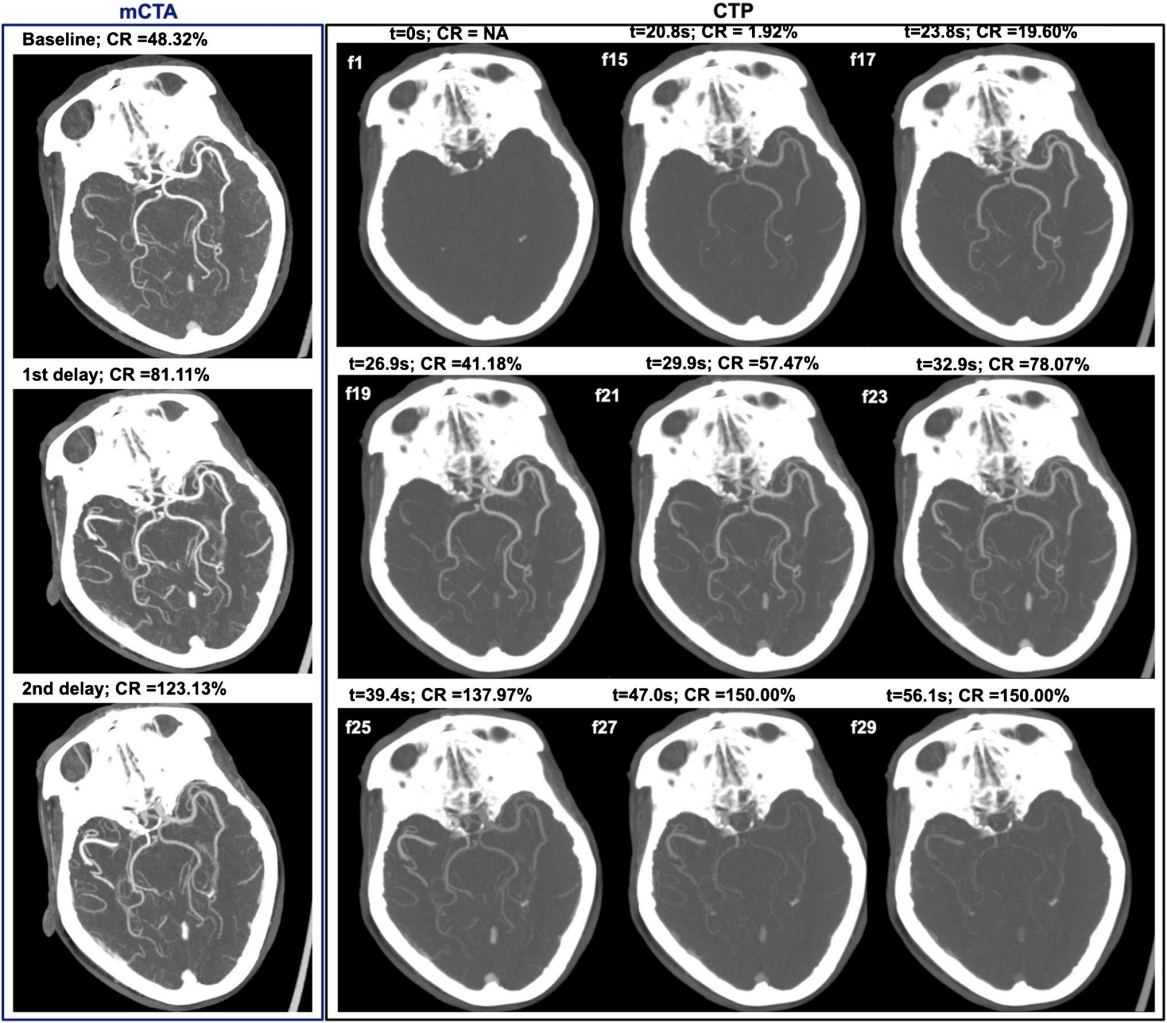
The example image of CR changing over time on both mCTA and CTP. On CTP part, “f” indicates the frame number of CTP images, “t” indicates the relative acquisition time. In this example, mCTA baseline corresponds approximately to f19 in CTP; mCTA delay 1 corresponding to f23; mCTA delay 2 corresponding to f25

To be able to relate the CR in mCTA to the CRs derived from CTP, and to investigate the contrast acquisition phase of mCTA volumes, the mCTA images were temporally aligned to time points in the CTP acquisition in the same patient. This temporal alignment method was based on the AV enhancement ratios from the mCTA and the CTP images, using the timing information on the delays between the three mCTA volumes. We determined the optimal temporal alignment by minimizing the overall absolute difference of AV enhancement ratio in the mCTA images and the three corresponding time points in the CTP images. In addition, normalized AV enhancement ratio curves for all subjects were aligned at the arterial peak and the mCTA acquisitions were aligned accordingly. Subsequently, the acquisition phase of the each mCTA image was determined based on the CTP-based acquisition phase definition as described below. Figure 2 shows the image features of paired mCTA and CTP acquisitions

### CTP-based contrast acquisition phase definition

**Fig. 3:**
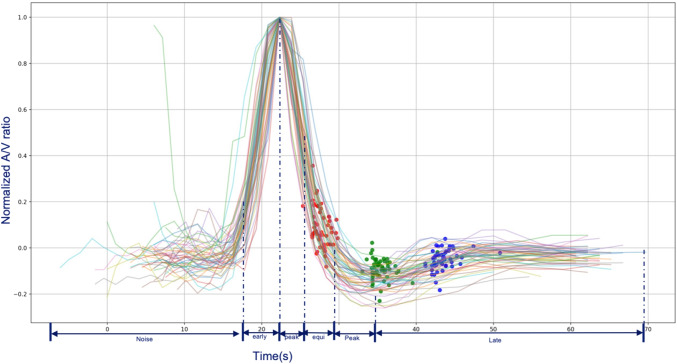
The result of mCTA to CTP alignment and contrast phase definition. Red dots represent the mapped mCTA baseline; green dots represent the mapped mCTA delay 1; blue dots represent the mapped mCTA delay 2. Each line represents a CTP image series

A contrast acquisition phase was defined based on the patient specific normalized AV enhancement ratio curve. The normalization was done by offsetting the equilibrium point (where AV enhancement ratio = 1) to 0 and dividing the result by the maximum value. Figure 3 shows the effect of this normalization for all included subjects, and also shows the positions of the temporarily aligned mCTA images. Subsequently, the contrast acquisition phase was defined using this curve as follows: early arterial phase: upslope in the ratio curve from 0.2 to 1.0; peak arterial phase: downslope from 1.0 to 0.5; equilibrium phase: downslope from 0.5 to 0; early venous phase: 0 to minimum ratio; late venous phase: minimum ratio to 0.

### Statistical tests

All values are reported as median and interquartile range (IQR). The Spearman correlation coefficient was used to analyze the relationship between the different CRs. To test the clinical relevance of choosing the optimal CR we assessed the association between the CRs and baseline ASPECTS and NIHSS with Spearman correlation coefficients and linear regression analysis. All statistical tests were performed using SPSS, IBM (version 26).

## Results

### Patients characteristics

In total, 335 patients were enrolled in Erasmus MC since January 2018 to March 2020. Baseline imaging was performed in the Erasmus MC on 106 patients. A CTP with slice thickness less or equal to 1.5 mm was available for 81 patients. Twenty-three patients were subsequently excluded because of vessel occlusions on both sides (n = 1), an incomplete CTP acquisition (n = 1), no baseline mCTA images (n = 7), large motion artifacts in the CTP acquisitions (n = 12), an NIHSS of 42 (n = 1) and baseline mCTA with DICOM header error (n = 1). The remaining 58 patients were included in this study. The median age was 74 years (IQR, 61–83 years), 33 were male, the median ASPECT score was 9, (IQR, 8–10) and the median baseline NIHSS was 16 (IQR 10–20). Fifteen patients had an occlusion in the intracranial ICA. The other patients had a middle cerebral artery (MCA) occlusion: 27 patients had a M1 segment occlusion, 15 patients had a M2 occlusion and the remaining patient had an M3 occlusion.

### Timing of acquisition and collateral status

The CR of the mCTA and CTP images at the predefined time points, and the corresponding time delay since contrast arrival are shown in Table 1. Huge differences exist between CR from CTP images obtained early after contrast arrival at the peak of CTP AV enhancement ratio (median 26%, IQR 12–52) and late in the venous phase at the CTP peak of venous enhancement (median 128%, IQR 88–171). The CR in the second delay mCTA volume had a median of 160% (IQR 119–241), indicating arrival of contrast via collateral vessels on the occluded side and diminished contrast in the healthy side due to normal blood flow.

**Table 1:** The characteristic of predefined Collateral Ratios (CR) and the corresponding time points in the aligned A/V curve

	Timepoint(in sec) Median [IQR]	CR(%) Median [IQR]	Observations in the acquision phases (n)				
			EA^a^	PA^b^	EQ^c^	PV^d^	LV^e^
Vessel volume peak at non-occluded side (*vol*_*max*)	7.6 [6.1, 7.6]	53 [39, 74]	−	5	53	−	−
Vessel volume peak at occluded side (*vol*_*occluded*_*max*)	9.1 [7.6, 10.6]	83 [66, 102]	−	−	26	31	1
Arterial peak (*arterial*_*max*)	6.4 [4.5, 6.1]	44 [31, 67]	−	27	31	−	−
Equilibrium (*equi*)	9.2 [8.3, 10.2]	78 [57, 92]	−	−	58	−	−
Peak venous (*venous*_*max*)	12.1 [10.6, 13.6]	121 [88, 171]	−	−	−	56	2
*r*_*av*_ peak (*av*_*max*)	3.0 [1.5, 3.0]	26 [12, 52]	7	51	−	−	−
mCTA baseline	8.1 [7.3, 9.0]	71 [46, 90]	2	8	48	−	−
mCTA delay 1	15.7 [14.9, 16.7]	125 [86, 183]	−		2	23	33
mCTA delay 2	23.8 [22.9, 24.8]	160, [119, 241]	−	−	−	3	55

The reference starting point (*t* = 0) is the contrast arrival time

^a^ *EA*, early arterial phase

^b^ *PA*, peak arterial phase

^c^ *EQ*, equilibrium phase

^d^ *PV*, peak venous phase

^e^ *LV*, late venous phase

**Fig. 4:**
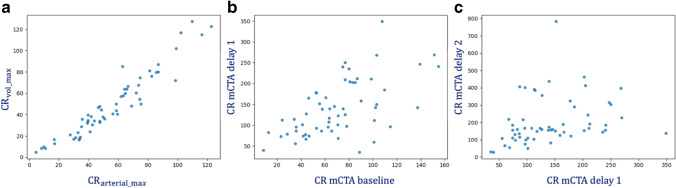
The correlation scatter plot. (**a**) The correlation between the CR at the peak of contrast volume in the non-occluded side ($CR_{vol\_max}$) and the peak of arterial enhancement ($CR_{arterial\_max}$). (**b**) The correlation between CR of mCTA baseline and delay 1. (**c**) The correlation between CR of mCTA delay1 and mCTA delay 2

The Spearman correlation coefficients between all CRs are shown in Table 2. The highest correlation between the CRs extracted from the CTP acquisition exists between the CR at the peak of contrast volume in the non-occluded side and the CR at the peak of arterial enhancement (r = 0.96; Fig. 4a). No correlations exist between the CRs in the mCTA acquisitions (Table 2 and Fig. 4b, c). When comparing the CRs from the CTP vs. mCTA acquisition, the highest correlations were found between CR from baseline mCTA and the time-resolved CR (r = 0.81) and the CR at the peak of arterial enhancement point (r = 0.78). The median time difference between baseline mCTA and arterial peak was 2.7s, IQR [1.7, 3.2].

**Table 2:** Correlation matrix of 10 proposed Collateral Ratios

	CR$_{vol\_occluded\_max}$	CR$_{arterial\_max}$	CR_*mip*_	CR_*equi*_	CR$_{venous_{m}ax}$	CR$_{av\_max}$	CR$_{mCTA\_baseline}$	CR$_{mCTA\_delay\_1}$	CR$_{mCTA\_delay\_2}$
CR$_{vol\_max}$	0.55	0.96	0.86	0.83	0.15	0.82	0.73	0.18	− 0.15
CR$_{vol\_occluded\_max}$	−	0.56	0.80	0.63	0.36	0.33	0.71	0.44	0.23
CR$_{arterial\_max}$	−	−	0.85	0.81	0.14	0.85	0.78	0.20	− 0.19
CR_*mip*_	−	−	−	0.85	0.30	0.61	0.81	0.41	0.13
CR_*equi*_	−	−	−	−	0.42	0.61	0.76	0.46	0.08
CR$_{venous_{m}ax}$	−	−	−	−	−	− 0.06	0.36	0.77	0.38
CR$_{av\_max}$	−	−	−	−	−	−	0.60	0.03	− 0.31
CR$_{mCTA\_baseline}$	−	−	−	−	−	−	−	0.59	− 0.04
CR$_{mCTA\_delay\_1}$	−	−	−	−	−	−	−	−	0.45

### mCTA to CTP time point alignment and phase classification

Fifty-six mCTAs out of 58 subjects were automatically temporarily aligned to the CTP acquisition based on the AV enhancement ratio curve. The two cases that failed were aligned manually. Figure 3 shows the aligned AV enhancement ratio curves with the temporally aligned mCTAs. The median of arterial to venous enhancement ratio difference from three acquisitions of mCTA to three time points of CTP was 0.2 (AV ratio ranged from 0 to 20). The median of the CR difference between baseline mCTA and temporally aligned CTP was 6.4% (IQR 2.4–12). The median of absolute CR difference between 1st delay mCTA and temporally aligned CTP was 16.5% (IQR 0–30). The median of absolute CR difference between 2nd delay mCTA and temporally aligned CTP was 0.2% (IQR 0–19). The acquisition phases of all predefined CRs are reported in Table 1. The acquisition phases of the CRs differ significantly from each other. Multiphase CTA baseline images were mainly acquired in the equilibrium phase (n = 48, 83%).

### Association of CRs and baseline clinical parameters

**Fig. 5:**
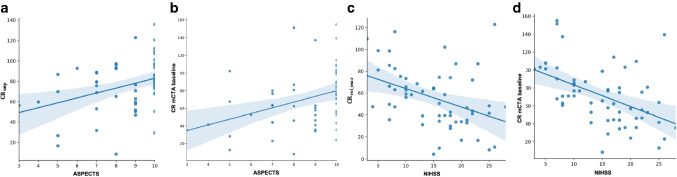
The linear regression plots. (**a**) The linear association between time-resolved CR (CR_*mip*_) and ASPECTS. (**b**) The linear association between CR of baseline mCTA and ASPECTS. (**c**) The linear association between CR at the volume peak of the non-occluded side (CR$_{vol\_max}$) and baseline NIHSS. (**d**) The linear association between CR of baseline mCTA and baseline NIHSS

Tables 3 and 4 list the correlations between the CRs at the proposed time points and baseline ASPECTS and NIHSS. The strongest correlation with baseline ASPECTS was found for CR determined from baseline mCTA (*β* = 0.36 (95%CI 0.11, 0.61)) and on time-resolved CTP (*β* = 0.33 (95%CI 0.07, 0.58)) (see Fig. 5a, b). The strongest association with baseline NIHSS was found for CR at baseline mCTA (*β* = − 0.48 (95%CI − 0.72, − 0.16)) and CR at volume peak of non-occluded side (*β* = − 0.40 (95%CI − 0.65, − 0.16)) (see (Fig. 5c, d).

**Table 3:** The association of the predefined collateral ratios with ASPECT score

CR variable (without threshold)	Spearman Correlation	Linear Regression
	*r* (p-value)	*β* (95%CI)
CR$_{vol\_max}$	0.37 (< 0.01)	0.32 (0.07, 0.58)
CR$_{vol\_occluded\_max}$	0.31 (< 0.01)	0.24 (− 0.03, 0.50)
CR_*mip*_	0.36 (< 0.01)	0.35 (0.10, 0.60)
CR$_{arterial\_max}$	0.38 (< 0.01)	0.33 (0.07, 0.58)
CR_*equi*_	0.34 (< 0.01)	0.28 (0.02, 0.53)
CR$_{venous\_max}$	0.01 (0.46)	0.06 (− 0.21, 0.33)
CR$_{av\_max}$	0.30 (0.01)	0.26 (0.05, 0.52)
CR$_{mCTA\_baseline}$	0.45 (< 0.01)	0.36 (0.11, 0.61)
CR$_{mCTA\_delay\_1}$	0.17 (0.1)	0.20 (− 0.06, 0.46)
CR$_{mCTA\_delay\_2}$	− 0.11 (0.22)	0.10 (− 0.17, 0.36)

**Table 4:** The association of the predefined collateral ratios variable with NIHSS baseline

CR variable (without threshold)	Spearman Correlation	Linear Regression
	*r* (p-value)	*β* (95%CI))
CR$_{vol\_max}$	− 0.40 (< 0.01)	− 0.40 (− 0.65, − 0.16)
CR$_{vol\_occluded\_max}$	− 0.24 (0.17)	− 0.12 (− 0.39, 0.14)
CR_*mip*_	− 0.42 (< 0.01)	− 0.37 (− 0.62, − 0.12)
CR$_{arterial\_max}$	− 0.44 (< 0.01)	− 0.39 (− 0.64, − 0.15)
CR_*equi*_	− 0.37 (< 0.01)	− 0.29 (− 0.55, − 0.04)
CR$_{venous\_max}$	− 0.07 (0.29)	− 0.02 (− 0.29, 0.25)
CR$_{av\_max}$	− 0.41 (< 0.01)	− 0.32 (− 0.58, − 0.07)
CR$_{mCTA\_baseline}$	− 0.53 (< 0.01)	− 0.48 (− 0.72, − 0.25)
CR$_{mCTA\_delay\_1}$	− 0.18 (0.10)	− 0.18 (− 0.44, 0.09)
CR$_{mCTA\_delay\_2}$	0.36 (< 0.01)	0.35 (0.09, 0.60)

## Discussion

In this study, we investigated the time dependency of collateral assessment in patients with an intracranial arterial occlusion. We defined objective CTP time points for CR extraction based on temporal curves of contrast enhanced vessel volume in the occluded and contralateral side, arterial enhancement and venous enhancement. We demonstrated that these CRs were significantly different from each other with an increase in CR in subsequent image volumes acquired during CTP. We subsequently compared the mCTA-derived CRs with the CRs obtained at predefined time points in the CTP and demonstrated that baseline mCTA correlates best with the predefined CTP CR at maximum arterial enhancement. To evaluate the potential significance of optimal CR assessment we related all CRs to baseline ASPECTS and NIHSS and demonstrated that baseline mCTA-derived CR had the highest associations.

Collateral status assessed on baseline CTA is an important parameter in clinical decision-making in patients with ischemic stroke due to intracranial large vessel occlusion and is an important predictor of outcome. Accurate, robust and reproducible assessment of CR is therefore crucial. The dependency of CR on the timing of image acquisition in relation to the contrast injection could be worrisome. This is especially true when automated analysis tools are able to provide a CR on a continuous scale instead of a semi-quantitative scale with a restricted number of grades (n = 4) as is the case with the current visual analysis. A CR could be two times larger when the CTA is acquired several seconds later. A CR could even be > 100% when the CTA is acquired in the venous phase. A previous study reported a higher prevalence of lower collateral grades in CTA scans acquired in the early arterial phase [4]. The lack of temporal resolution in sCTA, resulting in a highly susceptible to erroneous assessment of CR, has been identified before [6] and multiphase CTA has been proposed as reliable tool to provide a time-resolved collateral assessment [16]. With three CTAs in three different phases one may wonder which acquisition should be used for CR assessment, the authors solved this issue by proposing a new composite semi-quantitative scale with 6 grades which integrates information from the three phases in the mCTA. This collateral score for mCTA is different from the CR in our study, in which we assessed the quantitative CR per mCTA phase. Tong et al. have extracted one CR from CTP data after temporal fusion of the CTP images creating a time-resolved CR. This approach does not take into account the between patient variation in collateral recruitment which could have clinical consequences [26]. In our study, we reproduced this approach by generating an extra time-resolved CR_*mip*_.

The relevance of optimal timing of CTA for extraction of diagnostic information has also been reported for spot-sign detection in patients with intracerebral hemorrhage [27]. The authors proposed a classification for different acquisition phases, ranging from early arterial to late venous phase. The phase definition was based on absolute enhancement, expressed in Hounsfield units (HU), in arterial and venous structure. The dependence of HU on the voltage of the CT X-ray tube, and the dependence of the iodine concentration on the injection protocol (amount of contrast, concentration and injection speed) as well as hemodynamic factors [28], makes this definition of the phases rather arbitrarily. We proposed a phase classification based on a normalized arteriovenous enhancement ratio curve and demonstrated that most patients have identical normalized curves allowing a more robust assessment of acquisition phase for sCTA, mCTA and CTP image volumes.

We investigated in which phases the mCTAs were acquired and temporarily aligned the three mCTA volumes to the CTP volumes based on the arteriovenous enhancement ratio. Baseline mCTA was acquired mainly in the equilibrium phase, close to maximum arterial enhancement and therefore, the CR of baseline mCTA was highly correlated to the CR at maximum arterial enhancement. This consistent acquisition in the equilibrium phase, close to the maximum arterial enhancement is probably caused by the use of bolus tracking for timing of CTA in relation to contrast arrival and means that CR can be assessed reproducible from sCTA or mCTA, timed with bolus tracking.

The remaining question is which CR correlates best with the real status of brain perfusion and is optimal for clinical decision-making and outcome prediction. We demonstrated that several CRs are related to each other and could replace eventually each other. Others are less well correlated indicating that potentially more predictive information is available in the CTP derived CRs. To evaluate the potential significance of optimal CR assessment we determined the correlation between the CRs at the chosen time points to baseline ASPECTS and NIHSS and demonstrated that baseline mCTA-derived CR had the highest associations and was not outperformed by the other CRs. This could be explained by consistent acquisition of mCTA close to the peak of arterial enhancement.

Previous studies have compared the predictive power of mCTA-derived CR with sCTA-derived CR. Schreder et al., who extracted mCTA volumes from a CTP acquisition could not demonstrate a better outcome prediction compared to sCTA [29]. However, Wang et al. [13] investigated outcome prediction using single-phase and multiphase CTA images, also extracted from CTP acquisition. Considering the potential variation in hemodynamics between patients, the baseline mCTA and first delay mCTA was selected at the peak arterial phase and peak venous phase. The second delayed mCTA phase was defined by a time interval of 5.5 s after peak venous phase. They concluded that the mCTA composite collateral score performed better than single-phase collateral score in determining clinical outcome for patients with acute ischemic stroke. However, it is difficult to compare since the acquisition time of the single-phase CTA in comparison with mCTA images was unknown.

The results of the current study may have clinical implications as CTP is currently routinely performed in patients with acute ischemic stroke in some institutions, especially in the 6-24h time window. One may argue that an additional mCTA is not needed as for occlusion detection as demonstrated by Smit et al. [25] or collateral assessment as demonstrated in the current study. This study has several limitations. First, the dataset was obtained from small part of the MR CLEAN study and was collected in a single center. The generalization and validation of the proposed method are needed for the further assessment using multi-center data in a larger clinical study. Second, we use an automatic collateral scoring method that was developed for CTA on CTP images. The scoring method yields a relative value, where the quantification of the occluded side is normalized with the non-occluded side. We have assumed that this method is equally valid on CTP images, which may have a lower signal to noise ratio and contrast intensity compared to mCTA images. Last, we used CTP images to investigate the best time point for collateral scoring, based on associations with other baseline parameters. This study could not demonstrate a better association with baseline clinical (NIHSS) and imaging parameters (ASPECTS) for one of the CTP derived CRs over the mCTA derived CR. The small size of the study hampers strong conclusions. Finally, the choice for a specific CR should be based on an improved prognostic or predictive value, which should take into account also other predictors. Such an evaluation requires a large clinical study.

## Conclusion

We conclude that collateral status assessment is dependent on the timing of acquisition. mCTA with bolus tracking results in a consistent acquisition in the same phase close to the peak of arterial enhancement. This consistent acquisition in the equilibrium phase makes CTA derived CR assessment a robust and reproducible approach.

## Data Availability

The datasets generated during the current study are available from the corresponding author on reasonable request. The other datasets analyzed during the current study are available from MR CLEAN trial office (mrclean@erasmusmc.nl) on reasonable request

## Code availability

The code generated during the current study is available from the corresponding author on reasonable request.
